# The microbial conductor of cancer hallmarks: intratumoral microbiome as a multidimensional oncogenic modulator

**DOI:** 10.3389/fmicb.2025.1695187

**Published:** 2026-01-12

**Authors:** Yibin Wu, Mengyan Zhou, Yiqi Wan, Ke Wei, Shucheng Ma, Lijuan Cheng, Ting Lin, Lan He, Yingchun He, Fangliang Zhou

**Affiliations:** 1 Hunan University of Chinese Medicine, Changsha, China; 2 Hunan Provincial Engineering and Technological Research Center for Prevention and Treatment of Ophthalmology and Otolaryngology Diseases with Chinese Medicine and Protecting Visual Function, Hunan University of Chinese Medicine, Changsha, China; 3 Hunan Provincial Key Lab for the Prevention and Treatment of Ophthalmology and Otolaryngology Diseases with Traditional Chinese Medicine, Hunan University of Chinese Medicine, Changsha, China; 4 The First Affiliated Hospital, Hunan University of Chinese Medicine, Changsha, China

**Keywords:** cancer hallmark, immunotherapy, intratumoral microbiome, precision oncology, tumor microenvironment

## Abstract

The intratumoral microbiome, comprising diverse bacteria, fungi, and viruses residing within tumor tissues, is increasingly recognized as a multidimensional oncogenic modulator, acting akin to a “microbial conductor” orchestrating key cancer hallmarks. Its compositon exhibits substantial heterogeneity across individuals and is closely associated with the host immunity, the tumor microenvironment (TME), and therapeutic efficacy. Specific microbial species can “conduct” pro-tumorigenic processes by producing carcinogenic metabolites, dysregulating inflammatory signaling, or facilitating immune evasion. Conversely, other microorganisms may exert anti-tumorigenic effects by stimulating anti-tumor immunity or directly inhibiting cancer cell proliferation. Furthermore, the intratumoral microbiome can influence therapeutic outcomes by modulating the metabolism of chemotherapeutic agents or altering the efficacy of immunotherapies. Therefore, a deeper understanding of the intratumoral microbiome and its complex interplay with tumors holds immense potential to unravel fundamental mechanisms of cancer development and progression, while simultaneously revealing novel avenues for precision oncology strategies. This review outlines the biological roles of the microbiota in modulating the hallmarks of cancer hallmarks, summarizes current knowledge on its multidimensional interactions driving tumor progression, and discusses the translational potential of targeting or leveraging the intratumoral microbiome based on recent advancements. Future research integrating multi-omics profiling, spatial technologies, and functional validation will be essential for resolving methodological limitations and accelerating the clinical translation of microbiome-based interventions.

## 1 Introduction

**Figure 1:**
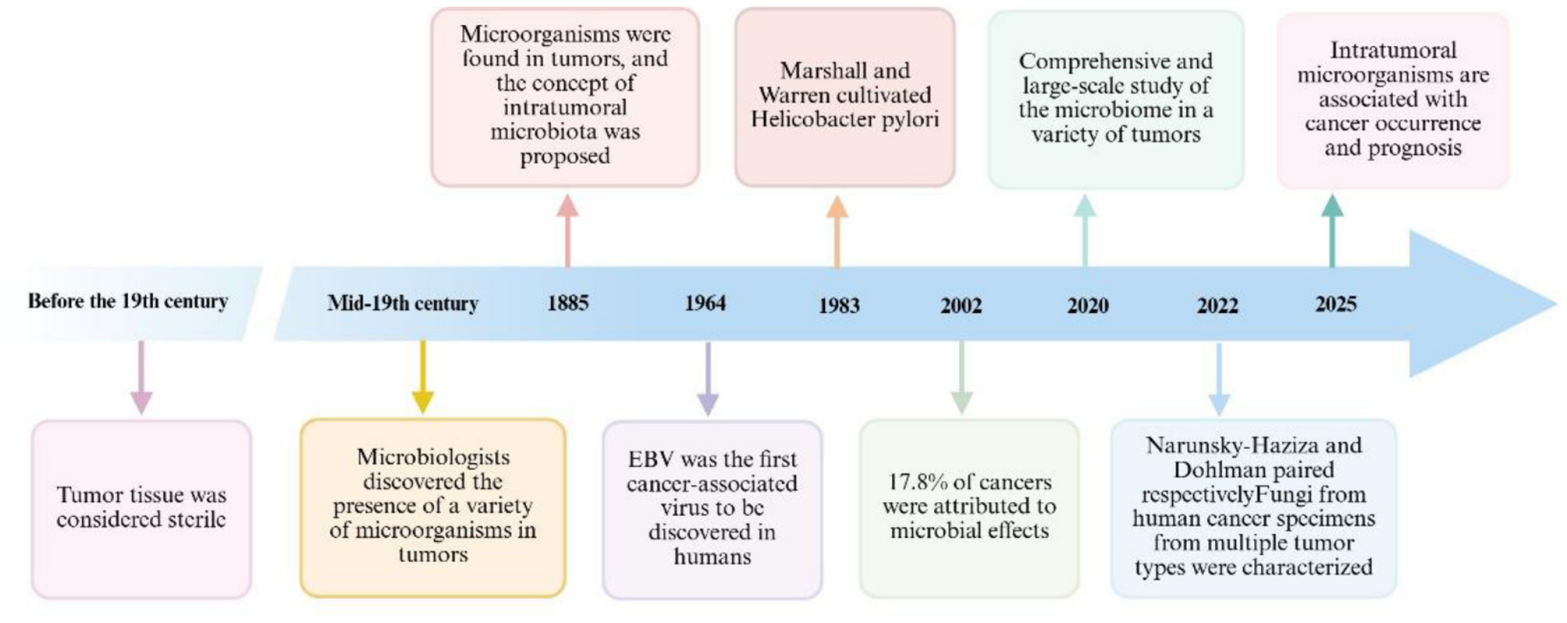
Milestone events of intratumoural microbiota. Review of the major discoveries regarding intratumoral microbiota. Created with BioRender.com.

**Figure 2:**
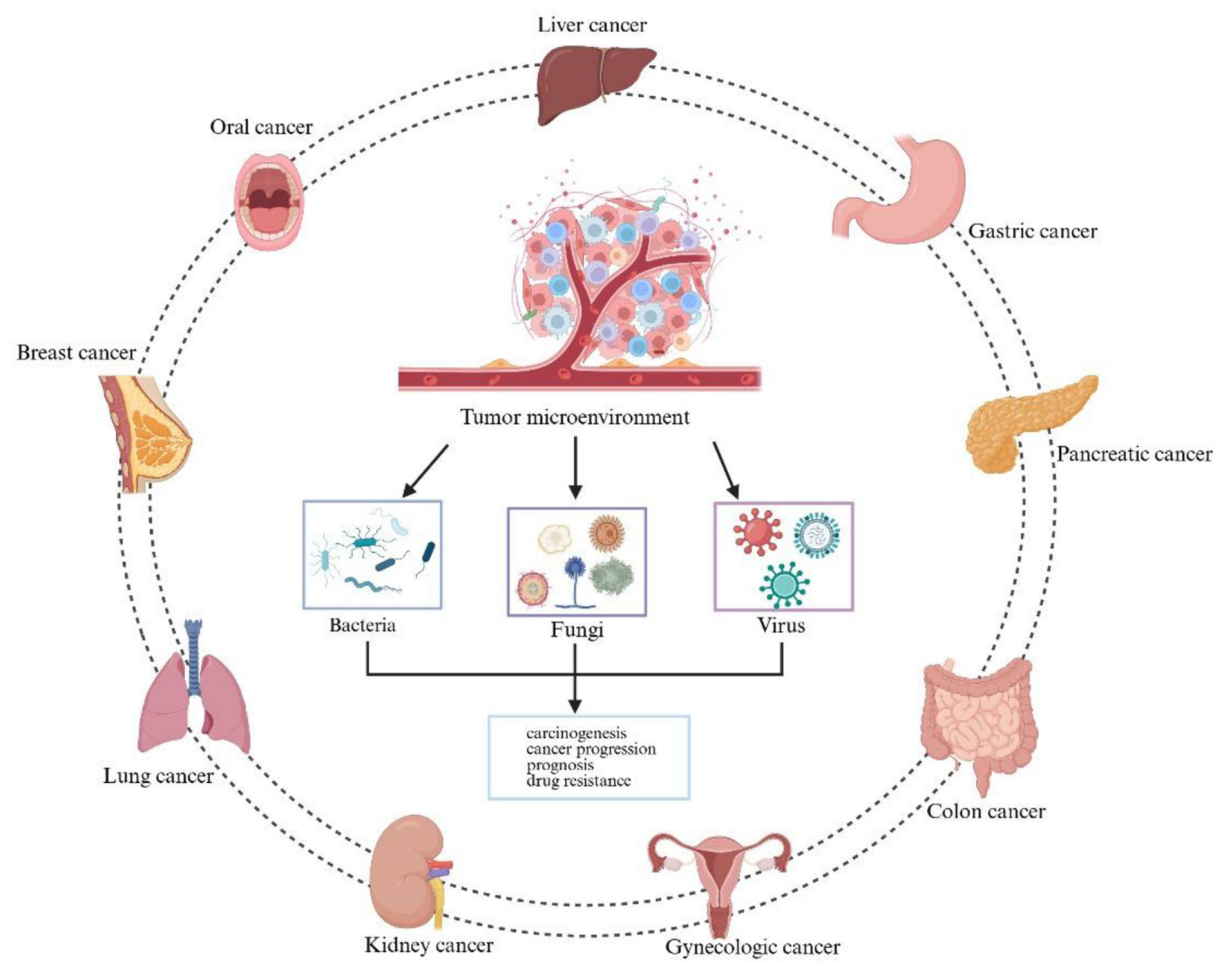
Intratumoral microbiota niches across cancer types. Microbiota have been detected in a wide range of solid tumors, including those of the liver, kidney, colon, gastric, lung, breast, oral and gynecologic cancer. The intratumor microbiome is considered a key contributor to carcinogenesis, tumor progression, prognosis, and therapeutic resistance. Created with BioRender.com.

The term “human microbiome” refers to the microbial communities—including bacteria, fungi, and viruses—that inhabit both the internal and external surfaces of the human body. Humans and these microorganisms coexist in a symbiotic relationship. These microbial communities are distributed throughout various anatomical sites, primarily colonizing mucosal tissues such as the skin, genital tract, oral cavity, and digestive system, with the gut serving as the primary reservoir for bacteria (Gao et al., 2022). Moreover, microbial presence has been identified in organs previously considered sterile, such as the thyroid, pancreas, and liver (Qu et al., 2022; Riquelme et al., 2019; Liu C. J. et al., 2021; Yuan et al., 2022; Sheehan et al., 2025). Each microbial community possesses a distinct composition and functional role that is closely associated with human health and disease (Campbell et al., 2020). So far, many important discoveries about intratumoral microbiota have been reported (Figure 1). Emerging evidence suggests that local microbiota significantly contribute to the tumor microenvironment in various cancer types, particularly in mucosal malignancies such as those of the lung, skin, gastrointestinal tract, and nasopharynx (Figure 2). Notably, the composition of intratumoral bacterial populations varies depending on tumor type. In a landmark study, Nejman et al. characterized the microbiomes of 1,526 samples from seven different human cancers, identifying significant differences in both the diversity and abundance of bacterial species between tumors and adjacent normal tissues (Nejman et al., 2020). A clinical study involving 507 lung cancer patients revealed that the abundance of intratumoral bacteria serves as a reliable prognostic indicator for stratifying the risk of malignant progression (Ochi et al., 2025). Intratumoral microorganisms exhibit substantial variation in both composition and abundance across different tumor types, which can influence various aspects of carcinogenesis, progression, and metastasis (Phipps et al., 2021). Clarifying the complex interactions between microorganisms and tumors may enhance the evaluation of current cancer therapies for individual patients and provide valuable insights into potential future treatment strategies (Riquelme et al., 2019; Nejman et al., 2020; Hu et al., 2025). This review proposes the core theoretical framework of the “microbial conductor” to systematically investigate how the intratumoral microbiota influences tumor initiation and progression. Instead of portraying microbial communities as passive bystanders, we regard them as active conductors that use metabolites and signaling molecules as their baton to systematically orchestrate the hallmarks of cancer, either promoting pro-tumorigenic dissonance or fostering anti-tumorigenic harmony. Building on this foundation, the review further examines the potential clinical applications derived from these insights. This integrated framework offers a novel theoretical perspective and serves as a valuable reference for future research.

## 2 Orchestrating cancer hallmarks: the microbial conductor as a framework

The conceptual framework of “cancer hallmarks” provides a fundamental paradigm for understanding the biological capabilities acquired during malignant transformation. This theory, pioneered by Hanahan and Weinberg (Hanahan, 2022; Hanahan and Weinberg, 2011, 2000), delineates core traits enabling tumor growth and dissemination, including: sustaining proliferative signaling, evading growth suppressors, resisting cell death, enabling replicative immortality, inducing angiogenesis, activating invasion and metastasis, reprogramming energy metabolism, evading immune destruction, tumor-promoting inflammation, genome instability and mutation, unlocking phenotypic plasticity, non-mutational epigenetic reprogramming, polymorphic microbiomes, senescent cells.

Critically, this systematic categorization of cancer hallmarks serves as an essential scaffold for investigating the multifaceted influence of the intratumoral microbiota. In this context, the microbiota acts as a conductor, orchestrating tumorigenesis through intricate microbial interactions, thereby modulating the hallmarks of cancer. Viewing these hallmark traits as primary targets of microbial regulation offers a powerful lens through which to dissect how resident bacteria, fungi, and viruses within the tumor niche function as multidimensional oncogenic modulators. The hallmarks framework allows researchers to methodically interrogate how specific microbial communities or their effector molecules directly or indirectly orchestrate these critical cancer-promoting processes—whether by fueling proliferative pathways, subverting immune surveillance, rewiring metabolism, or fostering a pro-metastatic environment. Therefore, based on the hallmarks of cancer, we delineate the core regulatory mechanisms through which the microbiota mediates tumor initiation and progression.

### 2.1 Marker 1: sustaining proliferative signaling

**Figure 3:**
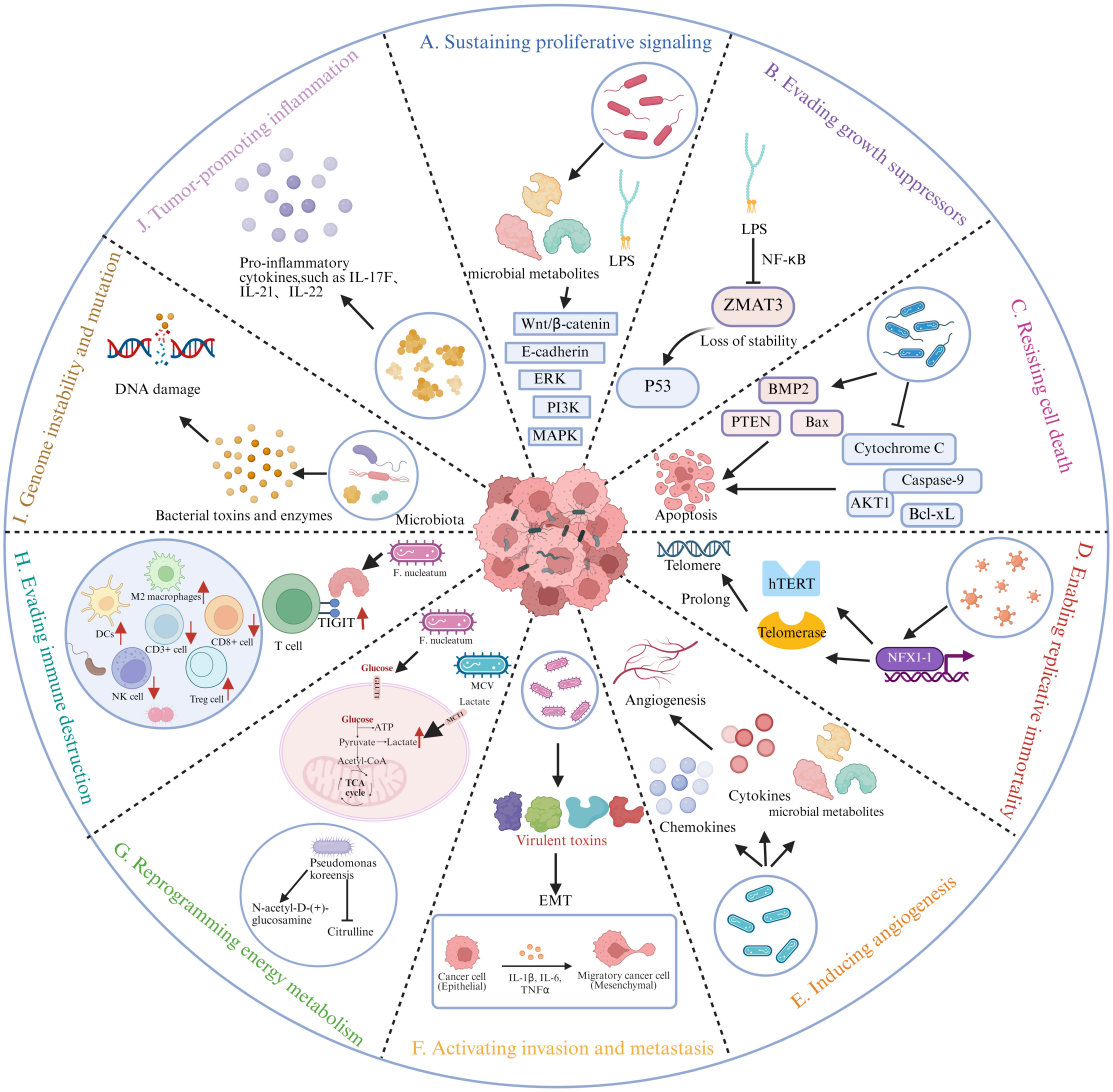
Relationship between intratumoral microbiota and the ten hallmarks of cancer. This schematic summarizes proposed mechanisms by which intratumoral microbiota influence ten recognized hallmarks of cancer: **(A)** Sustaining proliferative signaling, **(B)** Evading growth suppressors, **(C)** Resisting cell death, **(D)** Enabling replicative immortality, **(E)** Inducing angiogenesis, **(F)** Activating invasion and metastasis, **(G)** Reprogramming energy metabolism, **(H)** Evading immune destruction, **(I)** Genome instability and mutation, **(J)** Tumor-promoting inflammation. LPS, lipopolysaccharide; BMP2, bone morphogenetic protein 2; hTERT, human telomerase reverse transcriptase; EMT, epithelial-mesenchymal transition; GLUT1, glucose transporter 1; MCT1, monocarboxylate transporter SLC16A1. Created with BioRender.com.

One of the fundamental hallmarks of cancer is the sustained activation of proliferative signaling pathways (Hanahan and Weinberg, 2000). This ability enables cancer cells to autonomously drive their own growth, thereby circumventing the requirement for external mitogenic signals such as epidermal growth factor (EGF) and its receptor (EGFR). In an *in vitro* model, Tsay et al. demonstrated that exposing malignant bronchial epithelial cell lines to Streptococcus, Prevotella, and Veillonella resulted in the upregulation of the PI3K and ERK signaling pathways, thereby facilitating cancer cell proliferation (Tsay et al., 2018). Members of the PI3K family are involved in a broad range of cellular regulatory processes, including cell growth, proliferation, metabolism, migration, and secretion (Fruman et al., 2017). Importantly, several microbe-associated molecular patterns (MAMPs) and microbial metabolites can directly stimulate tumor cell growth (Figure 3A). For example, lipopolysaccharide (LPS) from Gram-negative bacteria activates TLR4–NF-κB/MAPK signaling in tumor cells, thereby promoting cancer cell survival and proliferation (Wang et al., 2013). Similarly, microbial metabolites also contribute to tumor progression. Short-chain fatty acids (SCFAs), such as acetate and propionate, have been shown to activate GPCR-mediated proliferative signaling pathways in certain cancer types (Mann et al., 2024). Furthermore, enterotoxigenic Bacteroides fragilis contributes to tumorigenesis through the secretion of a metalloprotease toxin known as Bacteroides fragilis toxin (BFT). BFT induces the cleavage of E-cadherin in colonic epithelial cells, leading to the release and subsequent nuclear translocation of the oncogenic protein β-catenin. Once translocated into the nucleus, β-catenin acts as a transcription factor that promotes epithelial cell hyperproliferation (Fulbright et al., 2017). The Wnt/β-catenin signaling pathway is a well-characterized oncogenic cascade in cancer, with activation of β-catenin leading to the transcriptional upregulation of genes involved in cell proliferation, differentiation, apoptosis, and migration (He et al., 2016; Zhou et al., 2022). Furthermore, Helicobacter pylori contributes to gastric carcinogenesis through the secretion of the cytotoxin-associated gene A (CagA) protein. Once translocated into host cells, CagA interferes with β-catenin signaling, thereby promoting tumor development (Buti et al., 2011). Salmonella facilitates tumor initiation and progression through the expression of AvrA, which activates the β-catenin signaling pathway and its downstream targets, including c-Myc and cyclin D1 (Lu et al., 2014).

### 2.2 Marker 2: evading growth suppressors

In addition to the hallmark ability to induce and sustain pro-growth signals, cancer cells must also circumvent powerful programs that negatively affect cell proliferation (Hanahan and Weinberg, 2000). Many of these programs are mediated by tumor suppressor genes. p53 is a potent tumor suppressor that inhibits tumor growth through multiple mechanisms. As a transcription factor, p53 coordinates the expression of target genes that promote cell cycle arrest, apoptosis, and DNA repair (Fridman and Lowe, 2003; Williams and Schumacher, 2016; Kastan et al., 1995; Kastenhuber and Lowe, 2017). ZMAT3 is a novel RNA splicing and homeostasis regulator and a key component of p53-mediated tumor suppression (Bieging-Rolett et al., 2020). The ZMAT3 gene encodes an RNA-binding protein that stabilizes p53 mRNA. Studies have shown that lipopolysaccharides from Klebsiella pneumoniae can trigger NF-κB axis-dependent suppression of ZMAT3 gene expression, which destabilizes p53 mRNA and impairs its tumor-suppressive activity (Aschtgen et al., 2022) (Figure 3B).

### 2.3 Marker 3: resisting cell death

During tumor evolution, cancer cells acquire diverse strategies to evade or suppress apoptosis, a fundamental defense mechanism against tumorigenesis (Roerden and Spranger, 2025). Studies have demonstrated that Porphyromonas gingivalis secretes nucleoside diphosphate kinase (NDPK), an enzyme that phosphorylates heat shock protein 27 (HSP27), thereby diminishing cytochrome c release and caspase-9 activation, ultimately inhibiting apoptotic cell death (Lee et al., 2018). In addition, Mycoplasma species can upregulate the expression of bone morphogenetic protein 2 (BMP2), thereby promoting cellular transformation and tumorigenicity, enhancing proliferation and migration, and concurrently suppressing apoptotic pathways (Jiang et al., 2008). Conversely, Escherichia coli Nissle 1917 (EcN), a probiotic strain, exerts antitumor effects in colorectal cancer by inducing apoptosis through the downregulation of AKT1 and Bcl-xL, as well as the upregulation of PTEN and Bax, thereby inhibiting cancer cell proliferation (Alizadeh et al., 2020) (Figure 3C).

### 2.4 Marker 4: enabling replicative immortality

Cancer cells have the ability to revert to a dedifferentiated, stem-cell-like phenotype, which enables them to divide uncontrollably and undergo metabolic adaptations, thereby facilitating survival under adverse conditions (Hanahan and Weinberg, 2000). The aberrant activation of telomerase is widely recognized as a key mechanism driving this hallmark phenotype. Under physiological conditions, telomeres progressively shorten with each round of DNA replication in normal somatic cells, ultimately triggering cell cycle arrest and cellular senescence (de Jesus and Blasco, 2013). However, telomerase is activated in all HPV-related cancers, thereby extending telomeric DNA and preventing cellular senescence, apoptosis, and crisis (Figure 3D). In cells expressing high-risk HPV E6, the elevated expression of NFX1-123 can alter cell growth dynamics and, over time, enhance the expression of human telomerase reverse transcriptase (hTERT) and telomerase activity. This telomerase-driven cellular immortalization may play a crucial role in the initiation and progression of HPV-related cancers (Vliet-Gregg et al., 2019).

### 2.5 Marker 5: inducing angiogenesis

Angiogenesis is a crucial component of tissue remodeling during tumor development, ensuring an adequate blood supply essential for sustained tumor growth and proliferation (Liu X. et al., 2025). While a direct causal relationship between bacteria and tumor-associated angiogenesis has not yet been definitively established, intratumoral bacteria have been shown to exacerbate inflammatory responses, leading to increased secretion of cytokines and chemokines that facilitate both angiogenesis and immune cell recruitment (Figure 3E). Emerging evidence suggests that microbiota-derived SCFAs may modulate angiogenic processes. Among them, butyrate—a prominent SCFA—influences multiple cellular pathways. Notably, decreased levels of sodium butyrate have been associated with enhanced angiogenesis (Castro et al., 2021). Research by Kuhn et al. demonstrated that Bacteroides species play a critical role in maintaining intestinal barrier integrity by stimulating intraepithelial lymphocytes to secrete interleukin-6 (IL-6). Although low levels of IL-6 are generally well tolerated and may contribute to mucosal homeostasis, elevated IL-6 levels can activate oncogenic signaling pathways that promote tumorigenesis by enhancing cellular proliferation, angiogenesis, and invasiveness, while concurrently suppressing apoptosis (Kuhn et al., 2018; Kumari et al., 2016). Recent studies have also highlighted the role of tumor-associated neutrophils (TANs) in modulating tumor progression, promoting angiogenesis, and regulating antitumor immune responses (Mantovani et al., 2011). Experimental data demonstrated that the number of tumor-associated neutrophils (TANs) in tumors from mice colonized with *Fusobacterium nucleatum* increased by an average of 13.4-fold compared to those in mice not exposed to the bacterium. Additionally, the population of CD11b^+^ myeloid cells was elevated by approximately 3.2-fold (Kostic et al., 2013). Differentiated CD11b^+^ myeloid cells, including macrophages, dendritic cells (DCs), and granulocytes, have been shown to play critical roles in facilitating tumor progression and angiogenesis (Coussens and Pollard, 2011). In gastric cancer, Lactobacillus has the potential to stimulate angiogenesis within the tumor TME through the production of significant amounts of N-nitroso compounds (Li et al., 2021).

### 2.6 Marker 6: activating invasion and metastasis

The epithelial-mesenchymal transition (EMT), a key developmental regulatory program, has been demonstrated to play a pivotal role in tumor metastasis (Allgayer et al., 2025). The virulence factor CagA of Helicobacter pylori is crucial to the bacterium's pathogenicity (Slater et al., 1999; Yamaoka, 2010). Studies have demonstrated that CagA induces the overexpression of miR-543, which directly targets SIRT1, a deacetylase. By interacting with SIRT1, miR-543 inhibits autophagy, thereby enhancing the EMT process and promoting the migration and invasion of gastric cancer cells (Shi et al., 2019). The downregulation of E-cadherin is widely recognized as a pivotal event in the initiation of EMT (Sommariva and Gagliano, 2020). Rubinstein et al. demonstrated that the virulence factor Fusobacterium adhesin A (FadA) interacts with E-cadherin, thereby enhancing the adhesion and invasion of Fusobacterium into epithelial and colorectal cancer cells (Rubinstein et al., 2013). Moreover, reduced expression of KCNJ11 elevates the levels of galactose-N-acetyl-D-galactosamine (Gal-GalNAc) on the surface of colorectal cancer cells, thereby facilitating the binding of the *Fusobacterium nucleatum* Fap2 protein to Gal-GalNAc. This enhanced interaction promotes bacterial adhesion and invasion of host cells, ultimately contributing to colorectal cancer initiation and progression (Yu et al., 2025). EMT enables transformed epithelial cells to acquire enhanced capabilities for invasion, resistance to apoptosis, and metastasis. The Methyltransferase3 (METTL3) has been shown to be positively associated with tumor cell proliferation, EMT, DNA repair, metastasis, and invasion. *Clostridium butyricum* inhibits METTL3 expression in colorectal cancer cells by upregulating G protein-coupled receptor 3 (GPR3), leading to the downregulation of vimentin and vascular endothelial growth factor receptor 2 (VEGFR2). This suppression attenuates EMT and vasculogenic mimicry (VM), ultimately inhibiting tumor metastasis (Zhang K. et al., 2023). Furthermore, studies have demonstrated that Clostridium perfringens enterotoxin (CPE) may serve as a potential therapeutic target for oral squamous cell carcinoma (OSCC). On one hand, CPE promotes OSCC cell proliferation, migration, and invasion by inducing the nuclear translocation of claudin-4 (CLDN4). On the other hand, CPE inhibits the phosphorylation of YAP1, thereby enhancing its expression and subsequently stimulating cancer cell growth and EMT, ultimately facilitating tumor metastasis. Thus, targeting CPE represents a promising strategy for OSCC therapy (Nakashima et al., 2020). *Fusobacterium nucleatum* activates the TLR4/AKT signaling pathway, downregulates Keap1, and upregulates NRF2, thereby enhancing the transcription of CYP2J2 and increasing the production of its metabolite, 12,13-EpOME, which in turn promotes EMT and metastasis in colorectal cancer. This signaling axis may serve as a potential biomarker and therapeutic target for colorectal cancer patients infected with *Fusobacterium nucleatum* (Kong et al., 2021). In human breast cancer cell lines, toxins secreted by Bacteroides fragilis within the tumor microenvironment induce a migratory and invasive phenotype, accompanied by elevated expression of the EMT-related transcription factors Slug and Twist. In tumor cells located in the mammary ducts, colonization by Bacteroides fragilis has been shown to promote enhanced metastasis to distant organs (Parida et al., 2021). A recent study demonstrated that tumor-resident intracellular microbiota within breast cancer cells promote metastatic colonization. Mechanistically, these intracellular bacteria modulate the host cell actin network and enhance tumor cell survival against fluid shear stress during circulation, thereby increasing metastatic efficiency (Fu et al., 2022) (Figure 3F).

### 2.7 Marker 7: reprogramming energy metabolism

Reprogramming energy metabolism describes the adaptive capacity of cancer cells to restructure energy acquisition and utilization pathways, particularly by enhancing aerobic glycolysis and metabolic flexibility, thereby enabling tumor growth and survival in metabolically hostile microenvironments (Hanahan, 2022; Hanahan and Weinberg, 2011). Emerging evidence indicates that intratumoral microbes actively participate in this process by modulating glycolytic flux, nutrient availability, and metabolite signaling pathways in cancer cells (Figure 3G). Distinct bacterial taxa have been identified across different tumor types, and their presence has been associated with alterations in tumor metabolic profiles and metabolic–immune crosstalk (Nejman et al., 2020; Xue et al., 2024). For instance, Pseudomonas koreensis has been found to be positively correlated with N-acetyl-D-glucosamine and negatively correlated with citrulline (Xue et al., 2024). Zheng et al. reported that *Fusobacterium nucleatum* stimulates glycolysis in colorectal cancer cells through the induction of ANGPTL4 expression (Zheng et al., 2021). Elevated ANGPTL4 levels subsequently facilitate colonization by enhancing GLUT1 expression and glucose uptake. *Fusobacterium nucleatum* also promotes the transcription of the long non-coding RNA ENO1-IT1 through the activation of the transcription factor SP1. The elevated ENO1-IT1 acts to recruit the histone acetyltransferase KAT7, leading to alterations in histone modification patterns at target gene loci, thereby facilitating enhanced glycolysis and tumorigenesis in colorectal cancer (Hong et al., 2021). *Fusobacterium nucleatum* has been shown to promote glucose transporter 1 (GLUT1) upregulation and lactate accumulation by activating the GalNAc-autophagy-TBC1D5 signaling pathway, thereby facilitating the progression of oral squamous cell carcinoma (Sun J. et al., 2023). *In vitro* studies indicate that Merkel cell polyomavirus (MCV) oncogene expression enhances the transcription of glycolytic genes, such as the monocarboxylate transporter SLC16A1 (MCT1), thereby promoting aerobic glycolysis, which is characteristic of malignant and rapidly proliferating tumor cells (Berrios et al., 2016).

### 2.8 Marker 8: evading immune destruction

Solid tumors have evolved mechanisms to evade immune surveillance, thereby avoiding elimination by T cells, B cells, macrophages, and natural killer (NK) cells (Agudo and Miao, 2025). The intratumoral microbiota further promotes immune evasion by inhibiting the infiltration of cytotoxic immune cells, impairing anti-tumor immune responses, and blocking the cytotoxic activity of immune cells against tumor cells, ultimately contributing to tumor initiation and progression (Tzeng et al., 2021) (Figure 3H).

Tumors colonized by *Fusobacterium nucleatum* can suppress the cytotoxic function of natural killer (NK) cells and the activity of tumor-infiltrating lymphocytes (TILs) through the interaction between the bacterial surface protein Fap2 and the human inhibitory receptor TIGIT, thereby shielding tumors from immune cell-mediated cytotoxicity (Mima et al., 2015). In colorectal cancer tissues, *Fusobacterium nucleatum* infection is inversely correlated with the abundance of CD3^+^ T cells. A reduction in CD3^+^ T-cell density may compromise immune surveillance and diminish anti-tumor responses, thereby facilitating tumor progression (Mima et al., 2015). However, Wang et al. revealed that *Fusobacterium nucleatum* can augment anti-PD-1 therapy in microsatellite-stable colorectal cancer, thereby overcoming tumor immune evasion (Wang X. et al., 2024). Dysbiosis involving enterotoxigenic Bacteroides fragilis (ETBF) induces the polarization of tumor-associated macrophages (TAMs) toward the M2 phenotype, thereby promoting immune evasion of colorectal cancer cells in mice. In germ-free mouse models, ETBF colonization activates the p-STAT3 signaling pathway, driving M2 macrophage polarization and fostering a chronic inflammatory and immunosuppressive microenvironment, ultimately accelerating colorectal cancer progression (Sui et al., 2020). Additionally, within the uterine microbiota, symbiotic Peptostreptococcus species may promote the expression of indoleamine 2,3-dioxygenase 1 (IDO1) by producing indole-3-acetic acid (IAA). IAA stimulates macrophages in endometrial tissue to secrete IL-10, which subsequently enhances IFN-γ production by CD8^+^ T cells. The increased IFN-γ levels further upregulate IDO1 expression in endometrial carcinoma (EC) cells, accelerating tryptophan (Trp) catabolism and kynurenine (Kyn) accumulation. Tryptophan depletion and Kyn-induced regulatory T cell (Treg) differentiation may suppress CD8^+^ T cell proliferation and cytotoxicity, thereby contributing to the establishment of an immunotolerant microenvironment within EC tumors (Wang Q. et al., 2024). Bile salt hydrolase (BSH)-expressing Bacteroides has been shown to promote colorectal cancer progression by elevating levels of unconjugated bile acids, which activate the β-catenin/CCL28 signaling axis and subsequently drive the accumulation of immunosuppressive regulatory T cells within the tumor microenvironment (Sun L. et al., 2023). Taken together, the available evidence suggests that microbial influences on the tumor microenvironment predominantly operate through the induction of immune tolerance and the exacerbation of T-cell exhaustion, thereby enabling tumor cells to escape effective immune surveillance.

Notably, some studies have also suggested that intratumoral microbiota can mediate immune activation and elicit anti-tumor immune responses (Shi et al., 2020; Overacre-Delgoffe et al., 2021; Zhang Q. et al., 2023). For instance, gut-resident Bifidobacterium species have been shown to preferentially colonize tumor sites and promote T cell–dependent anti-tumor responses by enhancing the STING/IFN-I signaling pathway in tumor-infiltrating DCs (Shi et al., 2020).

### 2.9 Marker 9: genome instability and mutation

The complex process of tumorigenesis is attributed to the continuous accumulation of genetic mutations in cancer cells (Martínez-Jiménez et al., 2020). DNA damage increases the mutation rate, ultimately contributing to tumor formation (Figure 3I). Infection with Helicobacter pylori can directly induce DNA double-strand breaks in gastric epithelial cells and enhance the activity of cytidine deaminase (CDA) enzymatic activity, leading to genetic damage and promoting gastric cancer through dysplasia (Lim and Chung, 2023). Metabolites such as indoleamine, produced by Morganella species, can increase DNA mutation rates and intestinal permeability, thereby accelerating the initiation and progression of colorectal cancer (Cao et al., 2022). Campylobacter species secrete cytolethal distending toxin (CDT), which induces DNA double-strand breaks and facilitates colorectal cancer development (Okuda et al., 2021). Human T-cell leukemia virus type 1 (HTLV-1), a retrovirus, can inhibit key mediators of DNA double-strand break repair—specifically, the E3 ubiquitin ligase and ubiquitin-conjugating enzymes (such as ring finger protein 8)—through its Tax protein, leading to increased genomic instability and the accumulation of oncogenic mutations (Giam and Semmes, 2016). *Fusobacterium nucleatum* can induce DNA damage and promote cell proliferation in colorectal cancer cells by activating the E-cadherin/β-catenin pathway in a FadA-dependent manner, accompanied by the upregulation of Chk2 expression (Guo et al., 2020). Moreover, recent findings demonstrate that KRAS mutations facilitate the intratumoral colonization of ETBF in colorectal cancer, and identify the miR-3655/SURF6/IRF7/IFN-β signaling axis as a critical regulatory pathway. This represents the first mechanistic evidence supporting this regulatory interaction (Chen et al., 2024). Escherichia coli strains carrying the pathogenicity island pks encode a set of enzymes that synthesize colibactin, a genotoxin that induces DNA alkylation at adenine residues and causes double-strand breaks in cultured cells (Pleguezuelos-Manzano et al., 2020). The tumor suppressor gene TP53 is the most commonly mutated gene in lung cancer (Robles and Harris, 2010). Studies have shown that in cases of squamous cell carcinoma harboring TP53 mutations, the abundance of Acidovorax species is significantly increased, suggesting that intratumoral microbial dysbiosis is closely associated with TP53 mutations and may act as a contributing factor in lung tumorigenesis (Greathouse et al., 2018).

### 2.10 Marker 10: tumor-promoting inflammation

The inflammatory microenvironment arising from latent inflammation serves as a prerequisite for cancer initiation (Denk and Greten, 2022). Numerous studies have demonstrated that intratumoral microorganisms can modulate cytokine production, trigger pro-inflammatory responses, and subsequently activate the NF-κB signaling pathway, thereby promoting tumor progression (Figure 3J). NF-κB is widely recognized as a central regulatory factor in cancer-associated inflammation (DiDonato et al., 2012). Hoste et al. reported that the skin microbiota mediates pro-inflammatory responses through Toll-like receptor 5 (TLR5) signaling, thereby influencing the progression of skin cancer (Hoste et al., 2015). Fusarium species have been shown to activate the Toll-like receptor 4 (TLR4)/myeloid differentiation primary response 88 (MYD88)/NF-κB signaling pathway in tumor cells, leading to increased expression of microRNA-21 (miR-21). The upregulation of miR-21 suppresses the expression of RAS protein activator like 1 (RASA1), thereby activating intrinsic RAS signaling, promoting the transcription of genes associated with cell growth and proliferation, and ultimately enhancing the proliferation and migration of colorectal cancer cells. Additionally, elevated levels of inflammatory cytokines, including IL-17F, IL-21, IL-22, and MIP-3A, have been observed in the serum of bacteria-infected mice. Goodwin et al. reported that the Bacteroides fragilis toxin (BFT) upregulates the expression of spermine oxidase (SMO), resulting in the production of reactive oxygen species (ROS) as byproducts of spermine oxidation, which subsequently cause DNA damage. They further observed that BFT-mediated upregulation of SMO increases the levels of SMO-dependent ROS and the DNA damage marker γ-H2A.X. The accumulation of DNA damage promotes the uncontrolled growth of epithelial cells, ultimately leading to the development of colorectal cancer. SMO, as a major source of bacterially induced ROS, is directly associated with tumorigenesis and may serve as a potential target for chemoprevention (Goodwin et al., 2011). In the colon, tumor-prone mice harboring enterotoxigenic strains of Bacteroides fragilis exhibit elevated IL-17 expression, increased epithelial DNA damage, accelerated tumor progression, and higher mortality rates (Dejea et al., 2018). SELE, a cell adhesion molecule involved in mediating inflammatory responses, exhibits significantly higher expression in tumor-adjacent normal tissues (NAT) than in liver tumor tissues. Recent studies have identified a significant inverse correlation between the relative abundance of Malassezia and SELE expression, suggesting a potential association between intratumoral Malassezia and the modulation of local inflammation (Shen et al., 2025).

### 2.11 Other signs

In 2022, Hanahan expanded the concept of cancer hallmarks by introducing four additional parameters: “unlocking phenotypic plasticity”, “non-mutagenic epigenetic reprogramming”, “polymorphic microbiomes”, and “senescent cells” (Hanahan, 2022). Specific microbial signatures have been detected in tissue and blood samples across various common cancer types, each associated with distinct microbial communities (Nejman et al., 2020; Mou et al., 2025; Liu S. et al., 2025; Zhang et al., 2025). These signatures have been utilized to distinguish healthy individuals from cancer patients, indicating their potential diagnostic value. A recent pan-cancer study examined the presence of cancer-associated fungi in 17,401 samples from 35 distinct cancer types. The findings revealed that, although fungal DNA and cells are present at low abundance in several common human cancers, they display specific community compositions across different cancer types. Distinct fungal species and their associated cellular compositions are strongly linked to specific cancer types (Narunsky-Haziza et al., 2022). Overall, tumor microbiomes are primarily composed of bacteria, with a relatively low abundance of fungi. The composition of the microbial community in adjacent normal tissues closely resembles that of tumor tissues. Some microorganisms are detectable across various tumor types, although their abundance varies among different cancer types (Liang et al., 2023). The gut microbiome plays a crucial role in maintaining host health by participating in various physiological processes, including the digestion and absorption of dietary nutrients, pathogen defense, the generation of bioactive metabolites, and signaling to the brain and other distant organs (López-Otín and Kroemer, 2021). Disruption of this bidirectional microbiota-host communication can lead to dysbiosis, which may subsequently contribute to aging and aging-related diseases, including cancer (Park et al., 2022). Moreover, research has further elucidated the connection between microorganisms and tumorigenesis by uncovering the epigenetic landscape, which includes mechanisms such as DNA methylation, RNA methylation, non-coding RNA (ncRNA) regulation, and histone modifications (Hong et al., 2021; Xia et al., 2020; Guo et al., 2024; Xu et al., 2022; Png et al., 2022). Currently, research on the role of the microbiome in unlocking phenotypic plasticity remains limited. A more comprehensive understanding of the involvement of intratumoral microbes in this process will help further elucidate the complexity, mechanisms, and clinical manifestations of cancer.

## 3 Application of intratumoral microbiome

**Figure 4:**
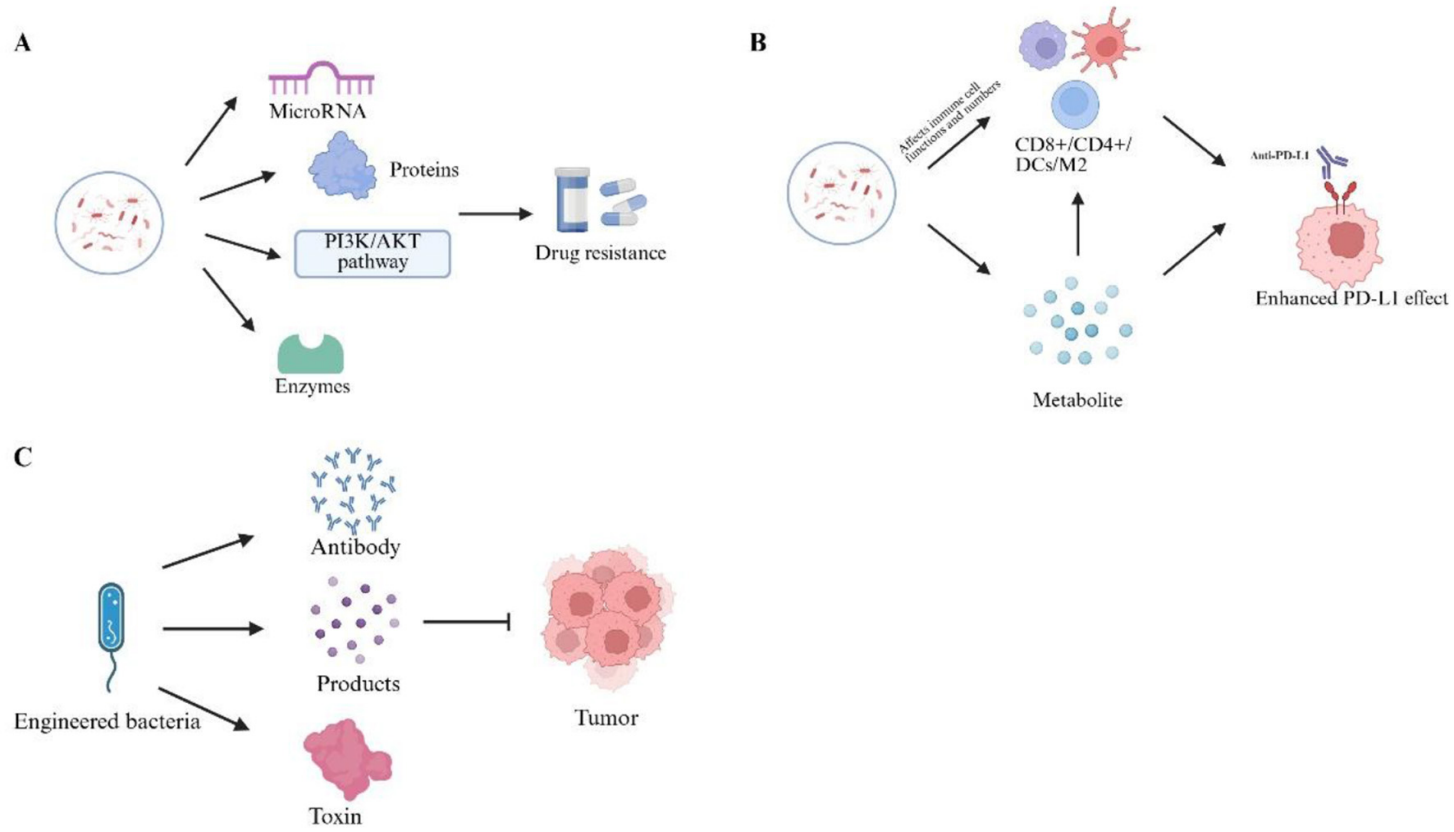
Prospects for the application of intratumoral microbiome. The intratumoral microbiome may **(A)** affect chemotherapy efficacy via enzymatic, metabolic, and signaling pathways, **(B)** modulate immune cells and checkpoints, and **(C)** serve as engineered vehicles to deliver therapeutic agents into tumors. Created with BioRender.com.

With the growing understanding of the tumor microbiome, it has become increasingly evident that microbes are not only key regulators of tumor progression but may also serve as novel targets for cancer therapy. In recent years, the potential roles of microbes in various therapeutic strategies, such as chemotherapy and immunotherapy, have garnered significant attention, particularly regarding their ability to modulate immune responses and overcome treatment resistance. Furthermore, the development of engineered microbes has paved the way for new possibilities in personalized cancer treatment (Figure 4). In this context, the tumor microbiome is emerging not only as an important component of cancer biology but also as a promising tool for cancer therapy, holding unique potential for clinical application.

### 3.1 Effect of intratumoral microbiome on chemotherapy

Resistance is a major cause of failure in cancer chemotherapy. Increasing evidence suggests that intratumoral bacteria may contribute to chemotherapy resistance by metabolizing anticancer drugs. Geller et al. analyzed human pancreatic cancer samples and found bacterial DNA in 76% of the samples, with γ-proteobacteria being the dominant group. The long-type isomer of cytidine deaminase (CDD) in these bacteria can modify the chemical structure of the chemotherapy drug gemcitabine, rendering it inactive and thereby promoting chemotherapy resistance in pancreatic cancer (Geller et al., 2017). Further studies have demonstrated that *Fusobacterium nucleatum* is enriched in colorectal cancer tissues of patients with chemotherapy relapse. It activates the autophagy pathway by downregulating miR-18a and miR-4802, which subsequently promotes chemotherapy resistance in colorectal cancer (Yu et al., 2017). A study by Lehouritis et al. found that Escherichia coli enhances the activity of tegafur while reducing the effectiveness of cytarabine, gemcitabine, and etoposide phosphate. In *in vivo* experiments using a mouse colon cancer model, they observed that, compared to the gemcitabine-only group, the gemcitabine + bacteria group exhibited a significantly larger tumor volume and lower survival rate, indicating that the antitumor activity of gemcitabine was diminished in tumors containing bacteria (Lehouritis et al., 2015). Further research suggests that microbiota dysbiosis contributes to chemotherapy resistance in tumors (Yang et al., 2021; Li et al., 2020; Gao et al., 2016; Liu and Zhang, 2022). For example, *Fusobacterium nucleatum* regulates the expression of endogenous LC3 and ATG7 proteins, thereby promoting the formation of autophagosomes, which contributes to chemotherapy resistance to 5-fluorouracil, cisplatin, and docetaxel (Liu Y. et al., 2021). Furthermore, butyrate produced by the gut microbiota has been shown to promote chemoresistance in colorectal cancer through activation of the PI3K/AKT signaling pathway (Xu et al., 2025). Studies have also shown that certain bacterial species are involved in the metabolism of anthracyclines. Streptomyces WAC04685 and Raoultella planticola have been described as inactivating doxorubicin through a deglycosylation mechanism (Westman et al., 2012; Yan et al., 2018). The combination of antitumor therapy and intratumoral microbiota may offer novel strategies to mitigate the occurrence of resistance. However, due to challenges related to regulation, variability, and safety, a significant gap persists between preclinical research and clinical application. Addressing these challenges and conducting rigorously designed clinical trials will be crucial for the development of microbiome-based adjunct therapies.

### 3.2 Effect of intratumoral microbiome on immunotherapy

In recent years, immunotherapy has become a critical component in cancer treatment. Immune checkpoint inhibitors (ICIs), such as those that block the interaction between programmed cell death protein 1 (PD-1) and its ligand, have shown promise in restoring the immune killing function of T cells. However, due to the limited activity of effector cells, particularly CD8+ T cells, most patients do not exhibit durable responses to this treatment (Miller et al., 2019). Emerging evidence demonstrates that the intratumoral microbiome critically shapes ICI efficacy by modulating CD8^+^ T-cell function, T-cell priming, cytokine signaling, and the overall immune microenvironment (Yang et al., 2024). For example, 16S rRNA gene sequencing analysis has demonstrated that Bifidobacterium plays a role in tumor control. Bifidobacterium activates the function of DCs within tumors, increasing the infiltration of CD8+ T cells and thereby enhancing the efficacy of anti-PD-L1 therapy to control tumor progression (Sivan et al., 2015). Streptococcus mitis inhibits the progression of gastric cancer by suppressing M2 macrophage polarization and infiltration and by modulating the composition of the intratumoral microbiota, thereby underscoring the critical role of the microbiome in shaping the tumor immune microenvironment (Yang et al., 2025). Cell-wall polysaccharides of Malassezia have been shown to activate the host complement cascade through mannose-binding lectin (MBL), a process that contributes to the progression of pancreatic cancer. Conversely, in pancreatic cancer, increased abundance of anaerobic bacteria (including Bacteroides, Lactobacillus, and Peptostreptococcus) is linked to diminished CD4^+^, CD8^+^, and CD45RO^+^ tumor-infiltrating lymphocytes, as well as an overall poorer prognosis (Abe et al., 2024). Microbial metabolites also provide functional inputs into T-cell regulation. Butyrate enhances CD8^+^ T-cell IL-12 responsiveness and IFN-γ production via ID2 upregulation, strengthening antitumor immunity (He et al., 2021). Lactobacillus reuteri has been shown to metabolize dietary tryptophan into AhR agonists, such as indole-3-aldehyde, which activate AhR signaling in tumor-infiltrating CD8^+^ T cells, thereby enhancing IFN-γ production, antitumor immunity, and responsiveness to immune checkpoint blockade (Bender et al., 2023). Moreover, recent studies have investigated and validated the intratumoral microbiome as a strong predictive biomarker for the response to Neoadjuvant immunotherapy combined with chemotherapy (Chemo-IM) in patients with early-stage triple-negative breast cancer (TNBC) (Chen et al., 2025). This strongly suggests that the microbiome plays a significant role in the efficacy of immunotherapy, with certain specific bacterial species being associated with enhanced therapeutic responses. However, the clinical application of intratumoral microbiota in immunotherapy continues to face significant challenges, including individual variability and technical limitations. Addressing these issues necessitates the development of more advanced analytical techniques and the establishment of standardized protocols to accurately evaluate the true impact of microbiota modulation on the efficacy of immunotherapy.

### 3.3 Anti-cancer effect of engineering bacteria

With the development of gene editing technologies, new methods for cancer treatment, including genetic engineering, have become feasible. Compared to traditional therapeutic approaches, genetic engineering offers significant advantages as a treatment strategy for cancer. Engineered bacteria can release specific products or induce certain reactions, thereby inhibiting tumor progression. Moreover, engineered bacteria can serve as vehicles for the targeted delivery of toxins, immune stimulants, or other therapeutic agents (Xie et al., 2022). Chowdhury et al. designed a non-pathogenic Escherichia coli strain capable of synthesizing CD47 nanobodies. These nanobodies self-accumulate and are released within the body, binding to the CD47 protein on cancer cells, thereby reducing their ability to evade immune responses and making them more susceptible to immune system attack (Chowdhury et al., 2019). Gurbatri designed a probiotic system that utilizes a stable lysis mechanism to regulate the release of PD-L1 and CTLA-4 nanobodies within tumors. This system enhanced T cell activation in mice, significantly inhibited lymphoma growth, elicited a distant effect, and improved survival (Gurbatri et al., 2020). Systemic administration of the highly potent anticancer agent tumor necrosis factor-α (TNF-α) can lead to high toxicity and severe side effects. Therefore, E. coli strain MG1655, as a tumor-targeting system, specifically produces TNF-α in CT26 mouse colon tumors, significantly inhibiting tumor growth (Murphy et al., 2017). Moreover, an attenuated Salmonella Typhimurium strain has been engineered to produce Vibrio vulnificus flagellin B specifically within tumor tissues in mice, thereby activating M1-like macrophages and suppressing M2-like macrophages through the TLR4 signaling pathway (Zheng et al., 2017). Despite the promising results of genetically engineered bacteria in cancer therapy, several challenges remain, including issues related to virulence, stability, uncontrolled proliferation, and the optimal drug delivery methods. Genetically engineered bacteria represent a promising new approach for the future of cancer therapy.

## 4 Conclusion and foresight

With the growing interest in understanding the relationship between the gut microbiome and tumors, the focus of research has gradually shifted toward exploring the impact of the tumor microbiome on tumor development and its effects on cancer therapy. Advances in the techniques for analyzing the gut and tumor microbiomes have significantly enhanced our understanding of the microbiome's influence on human health. However, research into intratumoral bacteria remains in its nascent stages. Importantly, host immune resilience represents an additional variable that further complicates the interpretation of microbial influences within tumors. Clinical evidence has shown that systemic factors—such as preoperative malnutrition-associated impairment of early immune recovery in cancer patients—can markedly reshape the host's baseline immune tone (Sun et al., 2025). These host-dependent differences may mask or modify the distinct effects of symbiotic vs. intratumoral microbes, making the biological boundaries between them even more difficult to define under current technical limitations.

Despite the growing interest in tumor-associated microbiomes, methodological limitations remain a central challenge in this field. Contamination represents a persistent concern in the analysis of low-biomass tumor samples; trace microbial signals originating from laboratory reagents, the environment, or even personnel can easily be misinterpreted as authentic tumor-resident microbes, thereby compromising the accuracy of conclusions (Mitchell et al., 2024). Recent studies have systematically reassessed microbial signals in cancer datasets, revealing that some reported microbial read counts may be significantly inflated as a result of contamination, thereby highlighting the critical need for rigorous validation and caution when interpreting sequencing data (Ge et al., 2025). Additionally, sample processing artifacts—such as differences in tissue preservation, lysis conditions, or extraction protocols—may alter the retention of microbial DNA, making direct comparisons across studies difficult. Current sequencing approaches also struggle to distinguish viable bacteria from dead cells or residual microbial DNA, limiting the functional interpretation of intratumoral microbes (Carr et al., 2021). Furthermore, reproducibility remains a major issue: library preparation procedures, and sequencing platforms can introduce systematic biases, leading to inconsistent microbial profiles across studies and even within the same laboratory. Consequently, independent cohort validation, cross-platform comparisons, and spatially resolved technologies—such as spatial transcriptomics and high-resolution imaging—are essential for ensuring the robustness and reproducibility of tumor microbiome research. Addressing these technical and methodological limitations will be critical for advancing the field toward greater precision and stronger clinical translational potential.

In addition to addressing these challenges, it is essential to elucidate the molecular mechanisms by which bacteria colonize and persist within the tumor microenvironment, as well as to characterize microbe–tumor interactions that influence tumor initiation, progression, and therapeutic response. Moreover, the translation of microbiome-based interventions into clinical practice requires the establishment of standardized protocols and reliable biomarkers for assessing the tumor microbiome and predicting treatment outcomes. In addition, determining how modulation of the intratumoral microbiome can enhance the efficacy of existing cancer therapies will facilitate the development of synergistic therapeutic strategies. Addressing these key challenges will ultimately advance the clinical application of tumor microbiome research and accelerate the development of microbiome-informed cancer treatments. Undoubtedly, the importance of the intratumoral microbiome in tumor biology is expected to play a key role in cancer research in the coming decades.
